# Nanocomposite Biopolymer Arboblend V2 Nature AgNPs

**DOI:** 10.3390/polym13172932

**Published:** 2021-08-31

**Authors:** Simona-Nicoleta Mazurchevici, Justina Georgiana Motaș, Mariana Diaconu, Gabriela Lisa, Nicoleta Monica Lohan, Mihai Glod, Dumitru Nedelcu

**Affiliations:** 1 Department of Machine, Manufacturing Technology, “Gheorghe Asachi” Technical University of Iasi, 700050 Iasi, Romania; simona0nikoleta@gmail.com (S.-N.M.); justinamotas@ymail.com (J.G.M.); 2 Department of Environmental Engineering and Management, Faculty of Chemical Engineering and Environmental Protection “Cristofor Simionescu”, “Gheorghe Asachi” Technical University of Iasi, 700050 Iasi, Romania; mdiaconu@tuiasi.ro; 3 Department of Chemical Engineering, Faculty of Chemical Engineering and Environmental Protection “Cristofor Simionescu”, “Gheorghe Asachi” Technical University of Iasi, 700050 Iasi, Romania; gapreot@ch.tuiasi.ro; 4 Department of Materials Engineering and Industrial Safety, “Gheorghe Asachi” Technical University of Iasi, 700050 Iasi, Romania; monica.lohan@yahoo.com; 5 Faculty of Medicine, Grigore T. Popa University of Medicine and Pharmacy Iasi, 700115 Iasi, Romania; mihai_glod@yahoo.com; 6 Mechanical Engineering Department, Technical Sciences Academy of Romania, 030167 Bucharest, Romania

**Keywords:** lignin-based polymer, thermal behavior, structure, composition, antibacterial behavior

## Abstract

Due to the pressing problems of today’s world, regarding both the finding of new, environmentally friendly materials which have the potential to replace classic ones, and the need to limit the accelerated spread of bacteria in hospitals, offices and other types of spaces, many researchers have chosen to develop their work in this field. Thus, biopolymeric materials have evolved so much that they are gradually becoming able to remove fossil-based plastics from major industries, which are harmful to the environment and implicitly to human health. The biopolymer employed in the present study, Arboblend V2 Nature with silver nanoparticle content (AgNP) meets both aspects mentioned above. The main purpose of the paper is to replace several parts and products in operation which exhibit antibacterial action, preventing the colonization and proliferation of bacteria (*Streptococcus pyogenes* and *Staphylococcus aureus*, by using the submerged cultivation method), but also the possibility of degradation in different environments. The biopolymer characterization followed the thermal behavior of the samples, their structure and morphology through specific analyses, such as TGA (thermogravimetric analysis), DSC (differential scanning calorimetry), SEM (scanning electron microscopy) and XRD (X-ray diffraction). The obtained results offer the possibility of use of said biocomposite material in the medical field because of its antibacterial characteristics that have proved to be positive, and, therefore, suitable for such applications. The thermal degradation and the structure of the material highlighted the possibility of employing it in good conditions at temperatures up to 200 °C. Two types of samples were used for thermal analysis: first, in the form of granules coated with silver nanoparticles, and second, test specimen cut from the sample obtained by injection molding from the coated granules with silver nanoparticles.

## 1. Introduction

Nanocomposites have attracted a great deal of interest in the biomedical industry, due to the fusion between biodegradable materials and metallic nanoparticles with an antibacterial role. Polymers with biodegradability properties are a viable alternative to plastics obtained from petroleum resources. While the physical and mechanical properties are equally good or even better, biopolymers are appreciated because they do not contribute to the decrease or the depletion of natural resources. The top-rated effect of biopolymers is the environmentally friendly behavior. The scientific approach chosen by the research team to obtain a thermoplastic material with antibacterial properties consisted of the inclusion of silver nanoparticles in the polymeric matrix of the base material, named Arboblend V2 Nature. This study aims to validate and demonstrate that a biopolymer-type material can be combined with metal nanoparticles, and in doing so improves its performance, thus becoming a material with antibacterial properties. The paper by [1] presents the research results on the fusion of a biopolymer in the form of granules, called Arboblend V2 Nature, with metal nanoparticles. According to the obtained results, combining the two materials by coating the biopolymer type granules with silver nanoparticles (AgNPs), using the vapor deposition process, was proven. The next step, after performing these experiments, represents the injection molding in the shape of the samples and the subsequent evaluation of the performance and properties of the new material obtained from Arboblend V2 Nature and silver nanoparticles. For the homogeneous mixture between the base material and the metallic nanoparticles, a two-phase process is required. The first part includes the coating of Arboblend V2 Nature granules with a thin layer of silver, followed by the melting of the mixture, which also helps to homogenize the metal nanoparticles with the thermoplastic material. The second part represents the actual injection and cooling of the obtained parts. Among the antibacterial additives used to obtain plastics with improved properties, including the ones which exhibit a bactericidal effect, those that have the ability to continuously release AgNPs are the most appreciated. It is considered that their effects are strongly linked to the stagnation of bacterial development, and in certain situations even eradicating them from the surface treated with silver ions [2,3,4,5]. Materials with antibacterial properties are suitable for medical applications that require additional protection against microbes, which is why the integration of AgNPs in a thermoplastic material has proven to be an inspired choice. It is well-known that silver nanoparticles are very toxic to a large number of bacteria, [6], including several antibiotic-resistant strains [7]. Previous studies confirming the antibacterial activity of silver nanoparticles indicate that the smaller they are (<10 nm), the better the antibiotic effect on the sample becomes [8]. The recent research on the manufacturing of nanocomposites has shown [9] that they have distinctive physical characteristics. They have been manufactured with a homogeneous distribution of silver nanoparticles in a polymer matrix, which has acquired antibacterial properties by fusing the two types of materials. So far, further analysis and studies are needed for the implementation and use of these antibacterial materials in public spaces which are most exposed to bacterial contamination, but also for their applicability in other areas. According to [10], in which the possibility of fusing a polymer (such as polypropylene) with silver nanoparticles was explained in detail, the results showed that the distribution of AgNPs in the polymer matrix can be obtained by injection molding. Before the melting of the polypropylene granules, they were coated with a thin film of silver by means of the vapor deposition process, which thus led to the homogeneous mixture of the AgNP in the polymer matrix, obtained through the melting of the material in the pre-injection step. However, it would be desirable if upcoming studies will show a comparison of other categories of nanoparticles immersed in the matrix of biopolymers, and their performance under certain environments. A method for increasing the applicability of antimicrobial metals is to incorporate them in the form of nanoparticles in a polymer/biopolymer, and thus producing a composite material. Depending on the subsequent applicability of the parts, the metals can be added both on the surface of a polymer and into the matrix [11]. Biopolymers are considered as an appropriate medium for the mixture and for stabilization of metallic nanoparticles, such as silver [12,13,14]. As per [15,16,17,18,19], it was shown that a higher concentration of AgNP is required to achieve higher antibacterial action. The antimicrobial effect in these applications is related to the release of nanoparticles from polymeric nanocomposites and their exposure to different bacterial cultures [20]. One of the strengths of AgNP is the low level of toxicity to human cells, instead negatively affecting bacteria and fungi leading to the inhibition of their reproduction [21]. However, according to recent studies in this area of expertise, there are existing health risks to consumers and workers which must be taken into account. Thus, it is considered that when silver ions released from AgNPs are inhaled morphomechanical and organelle perturbations can be caused, such as cell necrosis and triggered pulmonary inflammation [22,23]. The potential toxic effects were also observed on VBNC (viable but non-culturable) cells, and it was observed that when the cells are exposed to AgNPs, they fell into a VBNC state, instead of dying [24]. Furthermore, *Escherichia coli* (*E. coli*) bacteria develop a resistance to AgNPs but not to Ag (I) ions [25].

The novelty of this study is the newly made composite material itself in Arboblend V2 Nature (as base material) with AgNPs (as coating material for the granules). This allowed the obtainment of samples by injection molding, as well as the generation of a series of results on its thermal, morphological, structural and antibacterial behavior. This manuscript is important because it aims, in the last phase, to recommend the substitution of conventional plastics exhibiting bactericidal effects with the environmentally friendly equivalent, that has a similar performance, Arboblend V2 Nature with AgNPs. The replacement will mainly follow the applications in the medical industry because in this field it is necessary to combat the harmful effects of bacteria, but it may be employed in the food packaging industry as well. Furthermore, the ecological aspect is extremely important because the parts will be able to be collected, stored and subsequently degraded in various environments and/or solutions, depending on their behavior.

## 2. Materials and Methods

The selected material for the study was Arboblend V2 Nature, a lignin matrix biopolymer developed by a team of researchers from the Fraunhofer Institute for Chemical Technology (ICT), in collaboration with Tecnaro, Pfinztal (Tecnaro GmbH, Ilsfeld, Germany). According to the Tecnaro Biopolymer Company, the composition of Arboblend^®^, a completely biodegradable material, can include various constituents depending on the property profiles pursued by the manufacturer, such as: lignin, cellulose, bio-polyamides (bio-PA), polylactic acid (PLA), vegetable reinforcing fibers (flax, hemp, jute) and natural additives required for processing, [26]. This biopolymer was then coated with a 99.99% purity Silver (Ag) nanometallic layer using the Physical Vapor Deposition (PVD) method, with a VS-40 MITEC PVD sputtering equipment from the Department of Industrial Engineering, Tor Vergata University of Rome, Italy [1]. Two types of samples were obtained for the analysis: granules coated with silver nanoparticles (P1) and samples obtained by injection into the mold of granules coated with silver nanoparticles (P2).

The main parameters used to perform the coating were: deposition time, the amount of gas and working pressure.

The injection of the samples into the mold was performed on an SZ-600H injection molding equipment (SHEN ZHOU, Zhangjiagang, China) from the Laboratory of Fine Mechanics and Nanotechnologies, “Gheorghe Asachi” Technical University of Iasi, Romania. The main technological parameters were: injection temperature, injection pressure and cooling time.

In order to determine the thermal, structural and morphological degradation behavior, but also the antibacterial effect created by the silver layer, the following equipment and methods were used.

### 2.1. Thermal Analysis

Differential scanning calorimetry (DSC): The equipment used was NETZSCH differential scanning calorimeter DSC 200 F3 Maia type (NETZSCH-Gerätebau GmbH, Selb, Germany) with sensitivity: <1μW; temperature accuracy: 0.1 K; and enthalpy accuracy: generally <1%. The device was calibrated with mercury (Hg), Bismuth (Bi), Indium (In), Tin (Sn) and Zinc (Zn) standards. Samples weighing less than 30 mg were subjected to a temperature program consisting of heating from room temperature (RT) to 200 °C using a heating rate of 10 K/min, followed by cooling to RT using a cooling rate of 10 K/min. The experiment was performed in an atmosphere with argon (Ar) protection. DSC analysis was completed before the samples of these materials began to deteriorate. DSC thermograms were evaluated with Proteus software, using a tangent method for the determination of transformation temperatures. The transformation start temperatures (T_onset_), the peak temperature (T_peak_), the transformation end temperature (T_end_) as well as the amount of heat dissipated/absorbed were determined. The determination of the area was performed using a rectilinear baseline.

Thermogravimetric curves (TG), thermogravimetric derivatives (DTG) and differential thermal analyses (DTA) were recorded with Mettler Toledo TGA/SDTA 851 equipment. The mass of the samples subjected to thermal decomposition was less than 5mg. They were processed in an air atmosphere with a flow rate of 20 cm^3^/min. The study was performed in the 25–700 °C temperature range using a heating rate of 10 °C/min. The processing of thermogravimetric curves was performed with the STARe SW 9.10 software from Mettler Toledo (Columbus, OH, USA). In this case, several temperatures were determined: the temperature at which the thermal degradation begins in each stage (T_onset_), the temperature at which the rate of degradation in each stage is at its maximum (T_peak_), the temperature at which the thermal degradation ends in each stage (T_end_), as well as the mass percentage loss in each step, residue (W%).

### 2.2. Structural and Morphological Analyses

SEM structural analysis (scanning electron microscopy) and EDX (energy-dispersive X-ray spectroscopy) was performed on a QUANTA 200 3D electron microscope (FEI Company, Fremont, CA, USA). The SEM analysis was performed for both type of samples, P1 and P2. For the surface and cross-section analysis, micrographic maps of the samples were made. For SEM analysis, the images were obtained taking into account the following parameters: acceleration voltage of secondary electrons–30 kV, magnification power between (1000–5000)×; working distance, 15 mm; detector, LFD (large field detector) for the analysis of non-conductive samples (polymers, textile fibers, powders, etc.); tilt angle, 0°; the pressure inside the microscope chamber, 60 Pa.

Concerning X-ray diffraction analysis (XRD), given the fact that the results would not have been conclusive for the P1 sample that was coated with AgNPS, the aim was to identify the existence of specific Ag crystallization phases in the biopolymer composition. For this purpose, a sample of pure biopolymer (without silver nanoparticles) as well as a P2 sample obtained by injection into the mold of the granules coated with AgNPS were used to characterize the phase crystallinity of the biopolymer. Phase identification was performed by comparing the obtained data with those in the databases (96-901-3048) [27]. For X-ray diffractographic analysis, the X’Pert Pro MRD X-ray diffractometer was used, which is equipped with an X-ray tube with Cu kα anode, λ = 1.54 Å, Panalytical equipment (PANalytical, Almelo, the Netherlands), to which a voltage of 45 kV was applied, and the diffraction angle (2θ) varied between (5–90°). The data processing was performed with the help of X’Pert Data Collector, X’Pert High Score Plus and X’Pert Data Viewer programs (first software version number 3 and the second one version number 2.2 g, Malvern Panalytical, Malvern, UK), being finally rendered in the form of diffractograms in diffraction angle (2θ) coordinates and the absolute intensity of the maximum diffraction.

### 2.3. Antibacterial Analysis

The highlighting of the biocidal activity of the sample of Arboblend V2 Nature reinforced with silver nanoparticles was achieved by performing the submerged culture method, which consisted of bringing the test samples into contact with the test microorganisms, by culturing them in a liquid culture medium under aeration conditions and stirring for 72 h. In the experiments, two species of bacteria were used, namely *Staphylococcus aureus* and *Streptococcus pyogenes*, from the collection of the Microbiology Laboratory, Department of Engineering and Environmental Management of FICPM (Chemical Engineering and Environmental Protection “Cristofor Simionescu”).

#### 2.3.1. Biological Material

**Figure 1:**
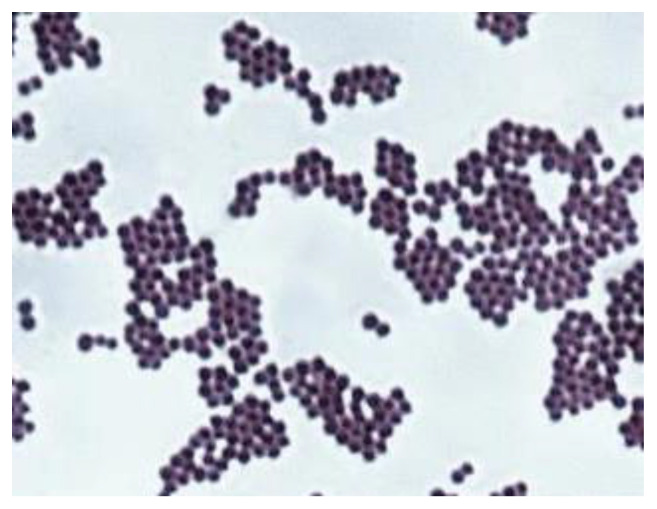
Gram’s staining of *Staphylococcus aureus* (1000×).

*Staphylococcus aureus* is a Gram-positive bacterium of spherical shape, shown in Figure 1, with the cells tending to accumulate in clusters after the division process. This bacterium is commonly found in the nose, respiratory tract and skin. It is often catalazo-positive, reduces nitrates and is an optional anaerobe that increases in the absence of oxygen.

**Figure 2:**
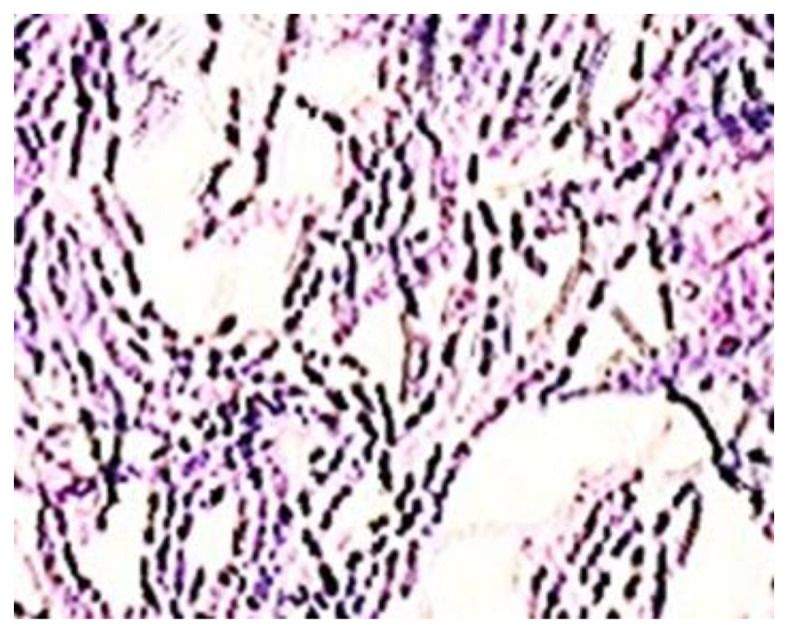
Gram’s staining of *Streptococcus pyogenes* (1000×).

*Streptococcus pyogenes*, shown in Figure 2, is a Gram-positive bacterium that grows in chains and causes many infections in humans, such as: pharyngitis, tonsillitis, scarlet fever, erysipelas, rheumatic fever, etc.

For the present paper, the submerged culture method was used, which involves contacting the test samples with the test microorganisms by culturing them in liquid culture medium under aeration and stirring conditions for 72 h. The following steps were performed.

Reactivation of bacterial species (*S. pyogenes* and *S. aureus*) on the Eugon Agar environment (LAB525) with the following composition (g/L) shown in Table 1.

The reactivation method of the species was as described. The ingredients were dissolved in 1000 mL of distilled water, and sterilized by autoclaving at 121 °C for 15 min. The pH is 7.0 ± 0.2. The medium was distributed in Petri dishes, and after solidification, it was seeded with bacterial strains. The bacteria development took place at a temperature of 37 °C, for 20 h, in order to obtain young cultures in an exponential growth phase.

**Figure 3:**
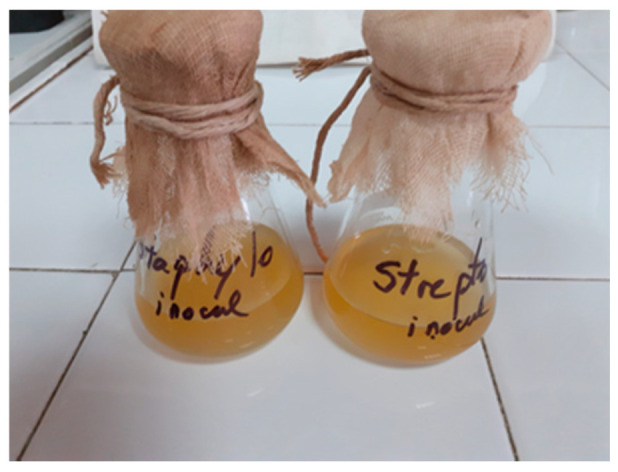
Erlenmeyer flasks with bacterial inoculum.

Obtaining the bacterial inoculum is completed by the seeding of a colonial fragment from each bacterial species in liquid culture medium (Eugon Broth—LAB 526) distributed in sterile Erlenmeyer flasks, shown in Figure 3. This culture medium had the same composition as above, except the gelling agent (agar). After sowing, the bacteria were left to grow for 18–20 h under aerated and stirred conditions at 37 °C.

The standardization of the cultures was realized by the method of serial dilutions. The procedure is as follows:

- A total 5 test tubes were prepared with 9ml of liquid culture medium for each bacterial tuple to be tested;
- Cell suspension of 1 mL was taken from the obtained inoculum, and was introduced into the first test tube with the liquid culture medium. It was homogenized very well and then serial dilutions are made until 10^−5^ dilution;
- In total, 5 sterile Petri plates were prepared, in which approximately (20–25) mL of agarized culture medium was poured. After solidification, the surface of the medium was inoculated with 0.1 mL of cell dilution suspension from 10^−5^ to 10^−1^. The suspension was uniformized on the surface of the culture medium by means of a sterile bacteriological loop, and then it was incubated for 24–48 h (as appropriate) for cell development and colony formation.

For the next stage, the dilution was chosen from those in which the colonies could be counted on the surface of the medium. This dilution will be used to seed the liquid culture medium, which is used to determine the biocidal activity of the Arboblend V2 Nature sample coated with silver nanoparticles. Dilution 10^5^ with 5 × 10^5^ CFU/mL (for *Staphylococcus aureus*) and 4 × 10^5^ UFC/mL (for *Streptococcus pyiogenes*) was chosen. UFC/mL = colony-forming unit/mL.

#### 2.3.2. Submerged Culture of Bacterial Strains in the Presence of the Test Sample

**Figure 4:**
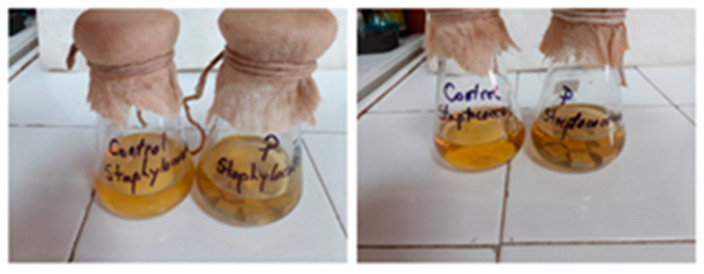
Submerged cultivation of bacterial strains.

Submerged cultivation (in liquid culture medium, Figure 4 has two advantages. On one hand, the bacterial cells are in permanent contact with the test samples present in their developmental environment, and on the other hand, due to the agitation process (200 rpm), the metal nanoparticles in the sample are released into the culture medium, and can thus influence the developmental process of test bacteria.

To eliminate the inherent variations that may occur when performing serial dilutions, it was preferred to operate with a single dilution of bacterial cells, namely 10^−5^, initially established at the inoculum for both bacterial species. Thus, the developed colonies on the surface of the agarized culture medium (Eugon Agar, LAB525) were transferred to 100 mL of liquid medium (Eugon Broth, LAB 526), homogenized very well, and then the medium thus inoculated was distributed in 2 sterile vials (50 mL), one for control and one in which 8 sample plates (1 cm^2^ × 8) were inserted for each bacterial strain tested.

**Figure 5:**
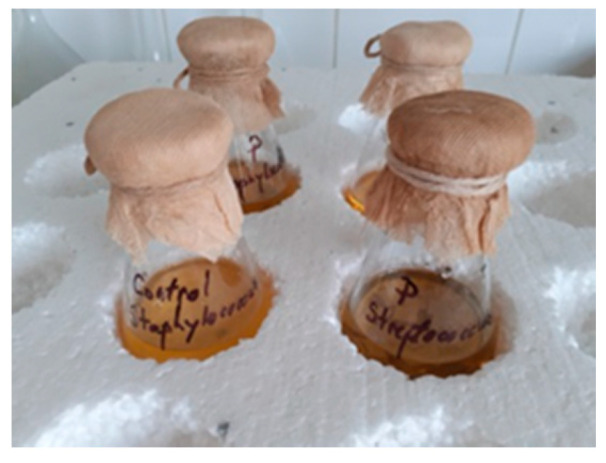
Incubated samples and controls.

Samples and controls were incubated, shown in Figure 5, for 72 h at 37 °C under aeration and stirring conditions (200 rpm).

## 3. Results

### 3.1. The DSC Analysis

The DSC analysis was performed in order to establish the physical transformations that take place during the heating of the samples, as well as to identify the temperatures at which they take place. Two types of samples were used: P1, sample in the form of a granule coated with silver nanoparticles (granule of Arboblend V2 Nature and AgNPs) and P2, sample cut from the test sample obtained by injection into the mold from coated granules with silver nanoparticles. The sample size was less than 5 mm. The P1 sample mass was 25.2 mg and the P2 sample mass was 26.0 mg.

During heating of both types of samples, three transformations were identified: two endothermic (first and last) and one exothermic (second) behavior, being similar to that reported in the literature for the base material [28].

**Figure 6:**
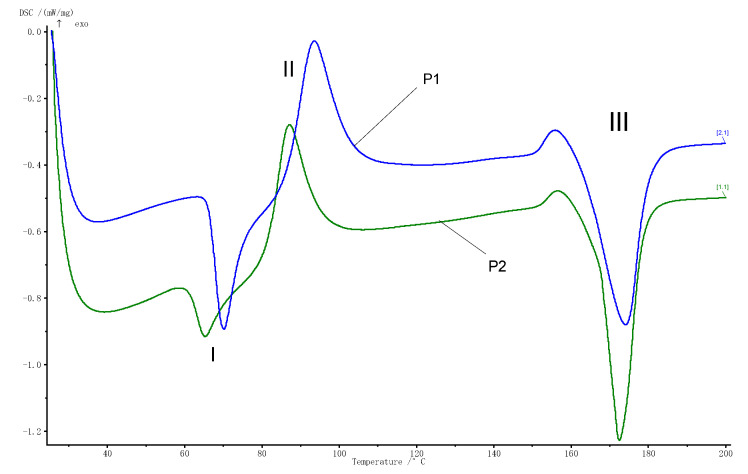
Highlighting the thermal behaviors of the tested samples: I, the first transformation; II, the second transformation; III, the third transformation; P1 (blue) and P2 (green) curves.

Figure 6 shows the variation in heat flow with the temperature recorded for the three phase transformations that take place during the heating of the samples. Arboblend V2 Nature granule coated with AgNPs, noted with P1; and the injected sample from Arboblend V2 Nature granules coated with AgNPs, noted with P2. The overlap of the two signals aimed to highlight the influence of the technological injection process, by melting the coated granules with AgNPs.

Both Figure 6 and Table 2 show shifts at lower temperatures for sample P2. The difference between the mass of the two analyzed samples is very small (P1, 26.0 mg and P2, 25.2 mg); therefore, the amounts of dissipated or absorbed heat (ΔH/m) can be considered as having significant importance [29].

From analyzing the calorimetric curves in Figure 6, the appearance of an endothermic maximum during heating is observed both for P1 located at 70.2 °C and for P2 located at 65.2 °C. It can be noticed that with the reheating of coated granules, the transformation temperature decreases by 5 °C, due to their injection in the mold. This decrease can be attributed to the incorporation of AgNPs in the Arboblend V2 Nature structure, but also to a new heating process of the material, which tends to structurally yield in the case of repeated heating’s [26,28]. According to the manufacturers, the material can be reused several times without losing its characteristics. However, as it is already known, the properties of a thermoplastic material are closely related to its thermal behavior. When the material is subjected to repeated heating, its structure, and, implicitly, its properties are progressively deteriorated, be they mechanical, physical or chemical. The first transformation (I) can be associated with a slow, monotropic transformation of solid-solid type of some metastable crystals [26], which takes place with low heat absorption, −5.66 kJ/kg in P1 and −12.68 kJ/kg in the case of the P2 sample. The variation of absorbed heat can be attributed to the incorporation of AgNPs into the biopolymer matrix.

The exothermic (II) transformation takes place at a temperature of 93.6 °C for P1 and at 87.2 °C for P2. The highlighted peak corresponds to the material crystallization, or it can be associated with the lignin reticular reorganization that is found in the Arboblend V2 Nature material structure [26].

The endothermic (III) transformation takes place with the absorption of a considerable amount of heat in the case of both analyzed samples, and is associated with the melting point of Arboblend V2 Nature material. The thermal maximum at which this transformation occurs for P2 is 172.6 ° C and P1 is 174.2 ° C.

### 3.2. The TGA Analysis

**Figure 7:**
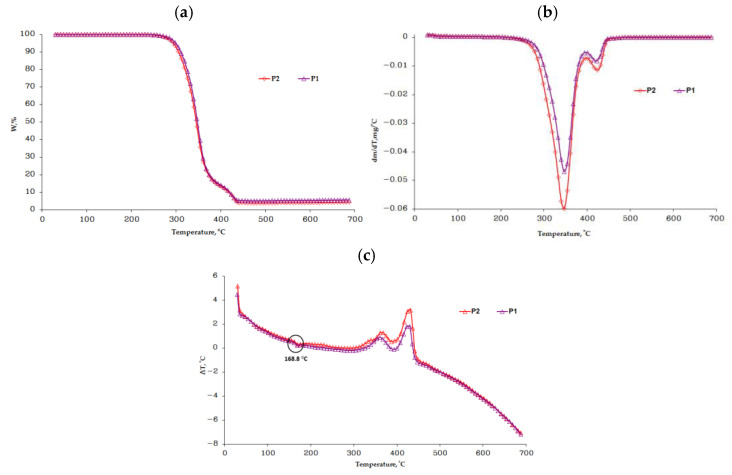
Thermogravimetric curves: (**a**) TG, (**b**) DTG and (**c**) DTA.

Considering the processing of the biocomposites by injection in the mold but also their potential applications in medicine, it is necessary to have a good thermal stability, both for the heating process required in the processing stages and to be able to subject them to sterilization processes without affecting their structure and properties. Figure 7 compares the thermogravimetric (TG), derived thermogravimetric (DTG) and differential thermal (DTA) curves.

From the thermogravimetric curves, the main thermal characteristics for the samples (granule and injected sample) were obtained, which are presented in Table 3.

The thermogravimetric analysis was performed in an atmosphere of synthetic air, with a heating rate of 10 °C/min in both types of samples, in order to detect the difference in behavior. For each analyzed sample, the thermal decomposition processes are as follows.

The granule from Arboblend V2 Nature coated with AgNPs (P1) showed two decomposition stages, the first in T_peak_ = 348 °C with a high mass loss of 83.80%, which is associated with the decomposition of the basic constituent of the material, lignin. This process consists in the formation of aromatic hydrocarbons, phenolics, hydroxyphenolics and guaiacyl-/syringyl-type compounds, etc. [30]. Another constituent present in the Arboblend V2 Nature granule was PLA, which also decomposes in this temperature range [31]. The decomposition of pure lignin and PLA in air atmosphere is performed completely without leaving any amount of residue up to a temperature of 500 °C [32,33]. In the second stage, with T_peak_ = 422 °C, a mass loss of 11.11% was observed, which was in addition to the thermo-oxidation of the carbon residue resulting from the decomposition of lignin and the degradation of another component of the material, probably an additive material such as resin or wax [26]. The residual mass of 5.09% was found at a 700 °C temperature, and is probably due to the presence of inorganic compounds in the material structure [34]. Most likely, a very small percentage of this residual mass can also be attributed to silver particles that have not even reached the melting point (961.8 °C).

The sample injected from Arboblend V2 Nature granules coated with AgNPs (P2), shown in Figure 7b, also decomposed into two stages, the first with T_peak_ = 346 °C with a mass loss of 84.44%, and the second at a temperature of 424 °C with a loss of 11.74%. The residue at the end of the TGA analysis, shown in Figure 7a was 3.82%, and this result was due to the presence of inorganic compounds in the material structure but also the presence of silver nanoparticles.

Overall, for the two analyzed samples, the thermal decomposition took place in two stages, with a higher percentage of mass loss in the first stage, in the (291–371 °C) temperature range. The main thermogravimetric characteristics, shown in Table 2, and the thermogravimetric curves (a), (b) and (c) in Figure 7 indicate that no significant changes occurred in samples P1 versus P2. There are only a few degrees of decrease in the onset temperature of the thermal decomposition for the injected sample. Slight variations also occurred for the amount of residue obtained at a temperature of 700 °C. In the case of the P2 sample, the amount of residue decreases by 1.27 percent, compared to P1. This difference can be attributed to the slight reduction in the thermal stability of the injected samples against the granules.

The DTA curves in Figure 7c show the melting temperature of the analyzed samples, which is around 168.8 °C, close to the values obtained for the two samples by the DSC technique.

### 3.3. Surface and Structure Analysis for P1 and P2 Samples

#### 3.3.1. SEM Analysis

**Figure 8:**
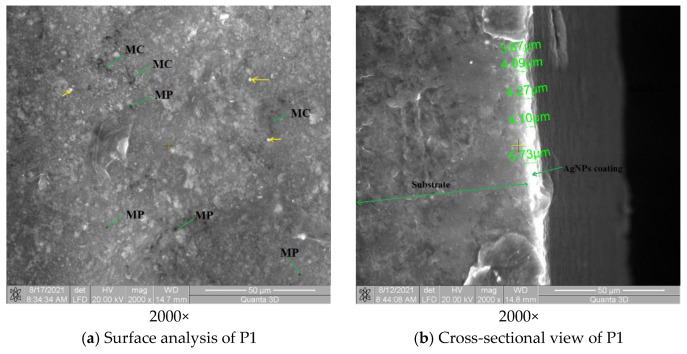

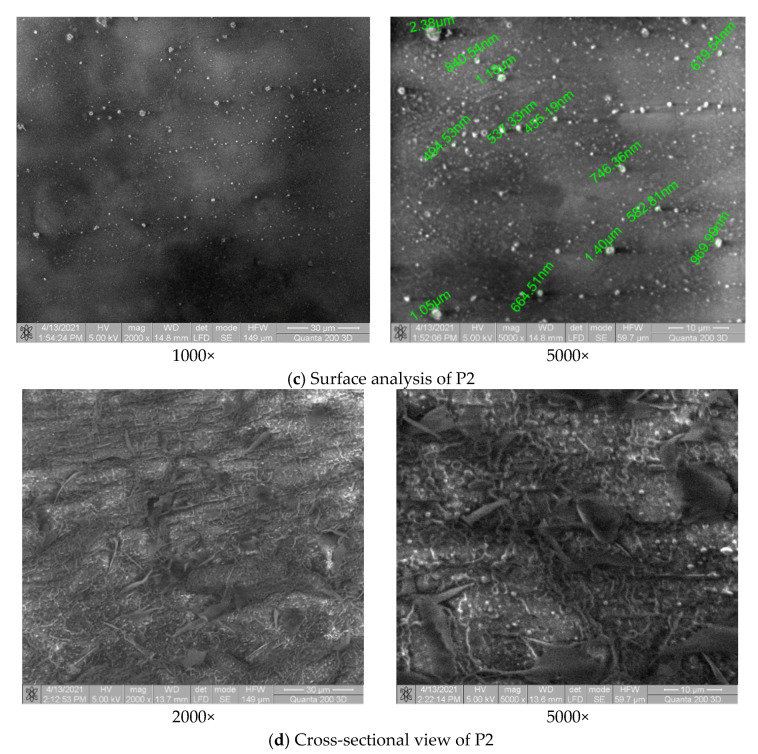
SEM analysis of the samples. (**a**) Surface analysis of P1; (**b**) Cross-sectional view of P1; (**c**) Surface analysis of P2; (**d**) Cross-sectional view of P2.

The surface analysis of P1, shown in Figure 8a, reveals a uniform layer deposition. Inside the coating are visible micro-cracks (noted with MC), micro-pores characteristic of PVD deposition process noted with MP and unmelted AgNPs (symbolized with a yellow arrow). The formed coating contains melted AgNPs.

The measurement of PVD AgNP coating thickness deposited on Arboblend V2 Nature granule is visible in Figure 8b. The existence of a relatively uniform deposited layer (AgNPs film, white line) was observed on the entire granule, with its average thickness being 4.37 µm. Furthermore, there was good adhesion between the polymer (substrate) and the coating (AgNPs), cracking or delamination at the interface not being visible.

The SEM analysis was performed both on the surface of the P2 sample and in its section, in order to demonstrate the structural homogeneity of the samples obtained by injection. Figure 8c shows the surface analysis of the injected samples, where the relatively uniform distribution of AgNPs nanoparticles can be observed, with their orientation being closely influenced by the injection direction in the mold. The injection was performed as a film at 90° along the sample length.

The cross-sectional structure of the sample, shown in Figure 8d, reveals a uniform spatial distribution of AgNPs; however, discontinuities in the internal structure of the sample can be observed, namely groups of nanoparticles that are bordered by polymeric structures. This means that the base polymer does not allow the formation of chemical bonds with silver.

#### 3.3.2. EDX Analysis

In order to identify the chemical structure of the injected sample from the coated granules with AgNPs, an Energy Dispersive X-ray Analysis (EDX) was performed, which provides information on the mass and atomic percentage of the chemical elements that are found in the P2 sample composition.

**Figure 9:**
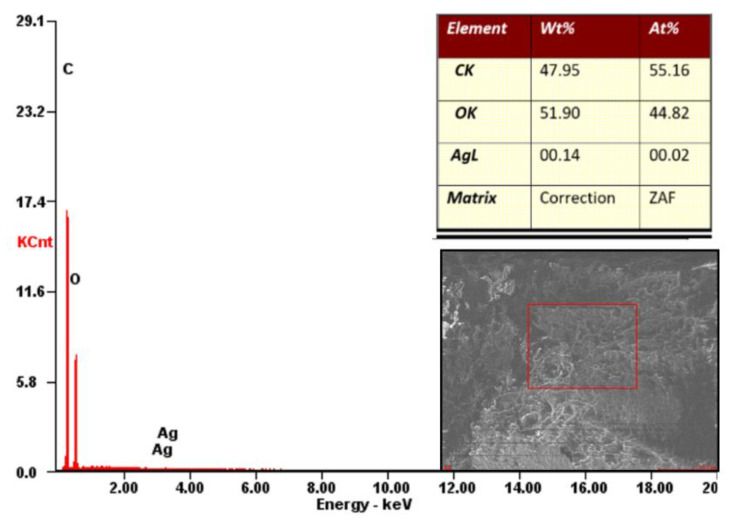
EDX spectroscopic analysis for P2 sample.

According to the obtained results from this analysis, shown in Figure 9, carbon and oxygen (in atomic and mass percentages) were distinguished as dominant constituents and were found in approximately equal proportions, C: 47.95%, O: 51.9%. Their presence in such a large quantity confirms the high degree of biodegradation, as they contain chemical elements that are found in abundance in the chemical structure of plants under different types of oxygen-carbon bonds (specific for cellulose, hemicellulose, lignin, lignin derivatives, etc. [26]). Furthermore, in the composition of the material, a very small amount, 0.14%, of silver (Ag) is found, which comes from the coating of biopolymeric granules with silver nanoparticles.

#### 3.3.3. XRD Analysis

**Figure 10:**
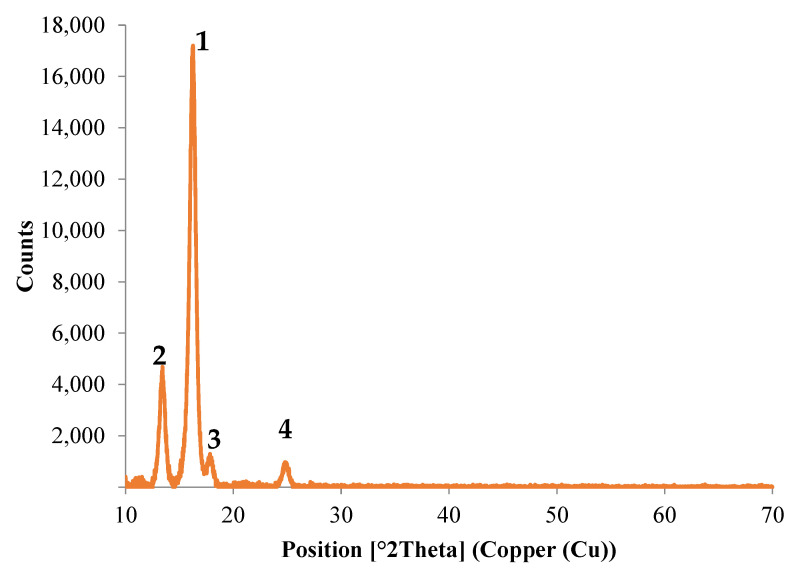
XRD analysis of the uncovered sample of Arboblend V2 Nature.

This analysis was performed in order to study the structure of the sample from Arboblend V2 Nature obtained by injecting granules coated with AgNPs, and to identify possible present crystalline phases. Based on these aspects, in order to obtain information on the chemical composition, XRD analysis was performed on both the pure biopolymer and the P2 sample. The pure Arboblend V2 Nature material showed a semicrystalline structure, as seen in Figure 10, with the following predominant peaks.

- There is a crystallization at an angle of 16.24° with an intensity of 17,163.61, which, according to the literature, can be associated with the presence of the composition of polylactic acid (C3H4O2, PLA) [35,36,37]. The presence of which is not surprising, since the manufacturers indicate it as a possible constituent of this biopolymer;
- The second major peak appears at an angle of 13.43° with an intensity of 4685.61, which corresponds to the lactone crystallization [38];
- Another significant peak was identified at 17.84°, and may correspond to polyvinylidene fluoride [39,40];
- The fourth peak is at an angle of 24.80° with a diffraction intensity of only 973.17, which corresponds to the area of cellulose crystallization. Similar results have been obtained by the authors of the papers in [41,42].

**Figure 11:**
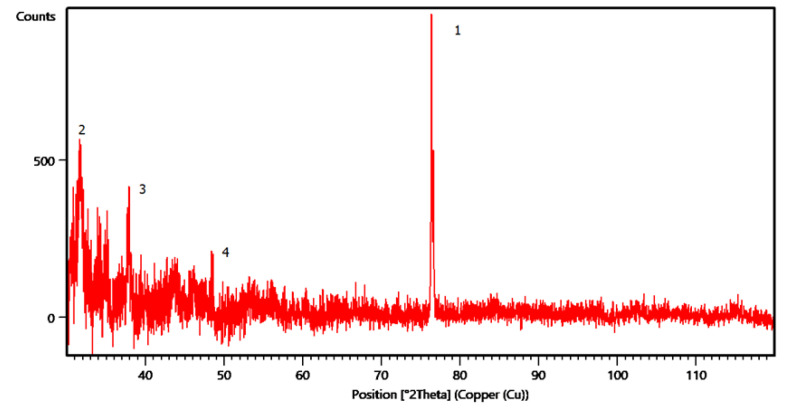
XRD analysis for P2 sample.

To identify the silver element in the sample injected with the composite material, a structural analysis was performed, at which the value of the 2θ angle was measured only for values above 30°, as according to the literature, no crystallizations of silver are visible under this angle. Figure 11 shows the structural analysis of the injected sample from granules coated with AgNPs, in which several peaks can be identified, and, according to the literature, correspond to the silver crystallization area. The predominant peak was recorded at an angle of 76.71° (marked with 1), followed in intensity by the peak at 31.42° (marked with 2), then the one at 37.81° (marked with 3) and finally the peak at 43.95° (marked with 4) [27,43,44,45]. The presence of these peaks that are specific to the silver crystallization certifies its incorporation in the biopolymeric structure.

### 3.4. Highlighting the Biocidal Activity of an Arboblend V2 Nature Sample Obtained by Injection into the Mold of Granules Coated with Silver Nanoparticles

After 72 h, in which the samples and controls were incubated at 37 °C, 1ml of cell suspension was taken from each sample and control for both test bacterial species. Serial dilutions were performed (10^−1^–10^−8^) in sterile saline. From dilutions 10^−8^–10^−4^, 0.1ml of cell dilution suspension was taken, of which the surface of the agarized culture medium (Eugon Agar, LAB525) distributed in Petri dishes was inoculated. After the uniformization of the cell suspension on the surface of the culture medium, the Petri dishes were incubated for 24 h at 37 °C for the microorganisms’ development.

**Figure 12:**
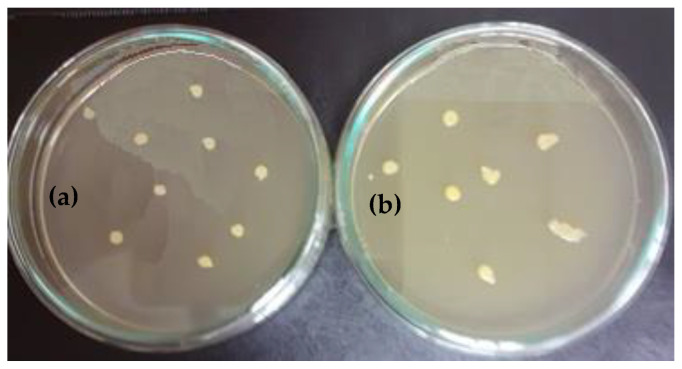
Number of UFC/mL resulting from submerged culture of *Staphylococcus aureus* (control (**a**) and sample (**b**)).

**Figure 13:**
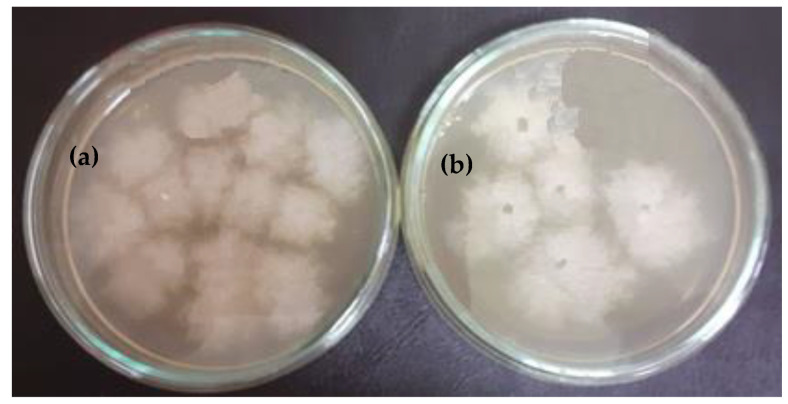
Number of UFC/mL resulting from submerged culture of *Streptococcus pyogenes* (control (**a**) and sample (**b**)).

The results obtained in this experiment are illustrated in the images from Figure 12 and Figure 13.

The following can be seen from the above figure.

- The bacterial species (*S. aureus*) developed very well in the control medium, obtaining 9 × 10^7^ UFC/mL, as seen in Figure 12a, which was a significant increase compared to the number of UFC/mL initially introduced in the experiment (5 × 10^5^ UFC/mL), see Figure 12b;
- In the sample coated with silver nanoparticles, an inhibition of the bacterium growth was observed, and the number of colonies that could be counted was at the 10^5^ (7 × 10^5^ UFC/mL) dilution. At 10^6^ to 10^8^ dilutions, no colony developed on the surface of the culture medium. However, a slight numerical increase was found compared to the amount of cells introduced in the experiment, which is probably due to the initial phase of development up until the diffusion of silver nanoparticles in the culture medium.

The following aspects can be seen in Figure 13: The number of colonies under control was 12 × 10^7^ UFC/mL, as shown in Figure 13a, while only 5 × 10^5^ UFC/mL (Figure 13b) were recorded in the sample. A rather pronounced inhibition of cell mass occurred in the presence of silver-coated samples. Thus, it can be mentioned that in the case of the control medium, and also of the sample at the dilution of 10^8^, no colonies were observed on the surface of the culture medium.

In conclusion, it can be stated that the tested samples had antibacterial action, leading to the inhibition of the test strains’ development.

## 4. Conclusions

The incorporation of silver nanoparticles (AgNPs) in the Arboblend V2 Nature biopolymer led to the following calorimetric, structural and morphological changes.

- From the DSC analysis point of view, the incorporation of nanoparticles led to a slight decrease in the transition temperatures;
- The thermogravimetric analysis indicated a slight reduction in the thermal stability of the injected samples against the granules;
- The peaks identified during the XRD analysis clearly demonstrate the existence of crystalline phases in the biodegradable thermoplastic material, examined both in its pure version and in the one containing silver nanoparticles. The identification of the peaks was only observed for those of significant intensity. Based on these, as well as thermal analysis and data from the literature, the appropriate hypothesis that the Arboblend V2 Nature and AgNPs sample has a semicrystalline structure was confirmed. Furthermore, the presence of silver nanoparticles in the biopolymer composition was confirmed by structural and morphological investigations: EDX, XRD and SEM.

The antibacterial resistance of the silver-coated Arboblend V2 Nature bioplastic material highlighted the possibility of preventing the colonization of bacteria, such as *Streptococcus pyogenes* and *Staphylococcus aureus* on the obtained samples. Therefore, this makes it possible to use the composite in the medical field (such as in clinical, dental, dermatological, cardiological applications) and in the food packaging industry, in order to prevent and reduce contamination of surfaces as much as possible. Regarding the second objective of the study, the gradual substitution of conventional plastics with biodegradable ones, it is fulfilled because both the antibacterial and structural, morphological characteristics of the Arboblend V2 Nature and AgNPs composite are comparable to those of nonbiodegradable polymers studied by other researchers, including silver polystyrene nanocomposites, Ag/PMMA nanocomposites, poly(methyl methacrylate), low-density polyethylene (LDPE), silver nanocomposites, polyamide/silver nanocomposites [46,47,48,49,50], and others.

## Figures and Tables

**Table 1:** Ingredients needed to reactivate *S. pyogenes* and *S. aureus* batteries.

**Ingredients**	**g/L**	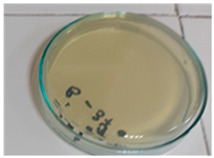
Tryptose	15.0	
Soy pepper	5.0	
Dextrose	5.5	
L-cysteine	0.7	
Sodium chloride	4.0	
Sodium sulfite	0.2	
Agar	15.0	

**Table 2:** Summary of data evaluation using Proteus software for calorimetrically characterized samples.

	Transformation	T_onset_ [°C]	T_peak_ [°C]	T_end_ [°C]	ΔH/m [kJ/kg]
**P1**	**I**	66.4	70.2	74.6	−12.68
	**II**	86.9	93.6	100.5	22.35
	**III**	162.3	174.2	179.6	−37.1
**P2**	**I**	61.3	65.2	69.8	−5.66
	**II**	81.6	87.2	94.4	23.57
	**III**	167.1	172.6	178.6	−38.85

The critical temperatures of the three transformations, T_onset_: starting temperature; T_peak_: middle temperature; and T_end_: finish temperature, were determined using the tangent method and the amount of dissipated/absorbed heat, ΔH/m, using a rectilinear baseline (highlighted also in Figure 6).

**Table 3:** Thermogravimetric characteristics.

Sample	Stage	T_onset_ [°C]	T_peak_ [°C]	T_end_ [°C]	W [%]	DTA characteristic	Residue [%]
P1	I	295	348	371	83.80	exo	5.09
	II	410	422	433	11.11	exo	
P2	I	291	346	371	84.44	exo	3.82
	II	413	424	442	11.74	exo	

T_onset_, the temperature at which thermal degradation begins at each stage; T_end_, the temperature at which the thermal degradation ends at each stage; T_peak_, the temperature at which the degradation rate at each stage is maximum; W%, percentage mass loss at each stage; residue, the amount of degraded sample remaining at a temperature above 700 °C.

## Data Availability

The data presented in this study are available on request from the corresponding author.
